# Familial risk of vasospastic angina: a nationwide family study in Sweden

**DOI:** 10.1136/openhrt-2023-002504

**Published:** 2023-12-06

**Authors:** Fabrizio Ricci, Behzad Banihashemi, Mirnabi Pirouzifard, Jan Sundquist, Kristina Sundquist, Richard Sutton, Artur Fedorowski, Bengt Zoller

**Affiliations:** 1 Gabriele d'Annunzio University of Chieti and Pescara, Chieti, Italy; 2 Department of Clinical Sciences, Lund University, Malmö, Sweden; 3 Center for Primary Health Care Research, Lund University, Malmö, Sweden; 4 Department of Cardiology, Imperial College School of Medicine, London, UK; 5 Department of Cardiology, Karolinska Institute and Karolinska University Hospital, Solna, Stockholm, Sweden

**Keywords:** GENETICS, CORONARY ARTERY DISEASE, Coronary Angiography, Angina Pectoris, EPIDEMIOLOGY

## Abstract

**Objectives:**

Vasospastic angina (VSA) is a complex coronary vasomotor disorder associated with an increased risk of myocardial infarction and sudden death. Despite considerable advances in understanding VSA pathophysiology, the interplay between genetic and environmental factors remains elusive. Accordingly, we aimed to determine the familial VSA risk among first-degree relatives of affected individuals.

**Methods:**

A population-based multigenerational cohort study was conducted, including full-sibling pairs born to Swedish parents between 1932 and 2018. Register-based diagnoses were ascertained through linkage to the Swedish Multigeneration Register and National Patient Register. Incidence rate ratios (IRRs) and adjusted HRs were calculated for relatives of individuals with VSA compared with relatives of individuals without VSA.

**Results:**

The total study population included 5 764 770 individuals. Overall, 3461 (0.06%) individuals (median age at disease onset 59 years, IQR: 63–76) were diagnosed with VSA. Of these, 2236 (64.61%) were women. The incidence rate of VSA for individuals with an affected sibling was 0.31 (95% CI: 0.24 to 0.42) per 1000 person-years compared with 0.04 (95% CI: 0.04 to 0.04) per 1000 person-years for those without an affected sibling, yielding an IRR of 7.58 (95% CI: 5.71 to 10.07). The risk of VSA for siblings with an affected sibling was significantly increased in the fully adjusted model (HR: 2.56; 95% CI: 1.73 to 3.79). No increased risk of VSA was observed in spouses of affected individuals (HR: 0.63; 95% CI: 0.19 to 2.09).

**Conclusions:**

In this nationwide family study, we identified high familial risk for VSA independent of shared environmental risk factors. Our findings indicate that VSA tends to cluster in families, emphasising the need to explore genetic and non-genetic factors that may contribute.

**WHAT IS ALREADY KNOWN ON THIS TOPIC**

- Vasospastic angina (VSA) is a condition associated with dynamic coronary artery obstruction and myocardial ischaemia. VSA is linked to both environmental and genetic factors, with smoking being the most significant environmental trigger, but uncertainties remain regarding familial risk.

**WHAT THIS STUDY ADDS**

- In this nationwide multigenerational cohort study, we observed a significant increase in VSA risk among siblings of affected individuals, independent of traditional cardiovascular risk factors, comorbidities and shared environmental factors, suggesting a strong hereditary component with genetic resemblance.

**HOW THIS STUDY MIGHT AFFECT RESEARCH, PRACTICE OR POLICY**

- The findings could shift the focus on genetic research for VSA, inform personalised treatment and encourage clinicians to include family history in risk assessments. Continued research is needed in multiethnic cohorts to uncover specific genetic factors and pathways involved in VSA pathophysiology.

## Introduction

Vasospastic angina (VSA), also known as Prinzmetal variant angina, is the clinical manifestation of myocardial ischaemia caused by dynamic coronary artery obstruction and sustained by a complex coronary vasomotor disorder resulting from endothelial dysfunction and non-specific hyper-reactivity of coronary vascular smooth muscle to vasoconstrictor stimuli.^1^ VSA has been associated with high rate of hospitalisations^2^ and major adverse cardiovascular events including myocardial infarction, syncope and sudden death.^3–6^ In Prinzmetal’s original description of variant angina, spontaneous episodes of rest angina were associated with transient ST elevation that promptly resolved with short-acting nitrates.^7^ Subsequently, these patients were shown to have inducible coronary artery spasm and the term VSA was introduced. The Coronary Vasomotor Disorders International Study Group (COVADIS) criteria for diagnosing VSA requires the presence of classical clinical manifestations, the documentation of myocardial ischaemia during spontaneous episodes and/or the demonstration of coronary artery spasm, to classify VSA into either ‘definitive’ or ‘suspected’.^8^ The 2023 Japanese guidelines^9^ refined the diagnostic algorithm with a focus on four items including symptom onset at rest (especially between night and early morning), significant diurnal variation in exercise tolerance, role of hyperventilation and effectiveness of calcium channel blockers.

Although recent advances in the understanding of VSA pathophysiology and improvements in diagnosis and treatment have been made, uncertainties still exist regarding the complex interplay of genetic and environmental factors contributing to the development of the disease. Indeed, although VSA was initially considered an acquired abnormality,^10^ previously referred to as ‘tobacco angina pectoris’ or ‘angine spasmo-tabagique’,^11^ substantial progress has been made to unravel underlying genetic factors. The available evidence highlights the roles of oxidative stress, inflammation, autonomic dysfunction and genetic susceptibility to VSA, as well as the presence of sex-specific differences and racial heterogeneity.^12^ Genetic mutations involving NO synthase, adrenergic receptors, serotoninergic receptors, angiotensin-converting enzyme and paraoxonase-I have been implicated in the pathogenesis of VSA.^13^ The C242T variant of the CYBA gene in men and the C634G variant of the interleukin-6 (IL-6) gene in women have also been linked to VSA. Additional variants of interest are the Ala370Ser variant of the ARHGAP9 gene and the ALDH2*2 genotype in East Asians. Despite the identified genetic associations,^11 14–17^ the environmental factor smoking remains the most significant adverse trigger for VSA, impacting several disease pathways related to these genetic variants and leading to substantial gene–environment interactions.^18^ Although smoking and nicotine are generally perceived as harmful, especially for those with strong genetic predispositions, they may sometimes yield paradoxical effects in certain individuals.^19^ This complexity suggests the existence of unknown patient-specific factors that could influence individual vasoactive response to these triggers.

Family risk studies can help to uncover familial patterns of disease and to differentiate the genetic and environmental components involved by comparing disease prevalence in siblings and spouses with and without affected family members.^20 21^ While family history is not generally believed a strong risk factor for VSA and genetic factors are considered as unlikely to be a major element in its pathogenesis,^22^ familial risk for VSA in a large population-based family cohort study has not yet been determined. Accordingly, we aimed to investigate the familial risk of VSA among siblings of individuals with or without a documented history of VSA in a nationwide family cohort based on data from Sweden. We hypothesised that there would be a higher risk of VSA among individuals with a documented family history of disease irrespective of shared environmental risk factors.

## Methods

### Study population

Patients were not involved in the design, or conduct, or reporting, or dissemination plans of our research. All data were provided by Statistics Sweden and the National Board of Health and Welfare for research purposes. Data were coded according to European Union law. The Regional Ethical Review Board in Lund, Sweden, approved this cohort study and waived informed consent as a requirement. This study followed the ‘Strengthening the Reporting of Observational Studies in Epidemiology ’ reporting guideline. We used the following Swedish national registers for data extraction^23–27^: the Swedish Multigeneration Register, which contains data on familial relationships and index persons born in 1932 and later and registered in Sweden in 1961 and later; the National Patient Register (NPR), which includes all hospital discharge diagnoses from 1964 to 2018 with nationwide coverage from 1987, and hospital outpatient diagnoses from 2001 to 2018; the population register and the total population register, which contain data on death date, if applicable, name change, marital status, family relationships, education and migration (the register has high coverage for nearly 100% of birth and death dates, 95% of immigration events and 91% of emigration events); and the Swedish Cause of Death Register, which provides date and cause of death from 1961 to 2018. The databases were linked together according to previously applied methods.^28 29^

In the Swedish Multigeneration National Swedish Register,^21^ we identified all pairs of full-sibling born in Sweden by Swedish-born parents. Thus, both biological parents were obligatorily known. Relative pairs with members who died or emigrated before 1997 or emigrated before the age of 17 years were excluded. Twins were included in the full-sibling group and no information regarding zygosity was available. The International Statistical Classification of Diseases and Related Health Problems, Tenth Revision (ICD-10 code I20.1) was used to identify VSA. We identified individuals with a diagnosis of VSA (ICD-10 code I20.1) registered between 1997 and 2018 from the NPR. In the database, all relative pairs were double-entered (ie, all full-sibling pairs as previously described).^28 29^ We allowed the same person to be included in more than one family relationship.

### Statistical analysis

Incidence rates were defined as the number of events divided by the person-time at risk. The familial incidence ratio between two incidence densities (rate in the exposed population divided by rate in those unexposed) gave the incidence rate ratio (IRR). VSA-free survival curves were constructed according to the Kaplan-Meier method to compare individuals with and without a documented family history of VSA. For the comparison of two curves, the log-rank test, resulting in a test statistic with a χ^2^ distribution and 1 df, was used. The ambiguity in unselected samples as to which sibling’s trait should be used as the dependent, and which as the independent variable is frequently resolved by using double entry. Each sibling entered twice in the data, and each member of a sibling pair provides once the dependent and once the explanatory variable. While the consistency of the regression estimates for heritability and environmental influences is not affected by double entry, the SEs of the coefficients are biased and need to be adjusted.^30^ In this study, adjusted familial associations between full-siblings were investigated using Cox proportional hazards model and were estimated using robust SEs. Results are reported as familial HRs with 95% CIs. Models were adjusted for year of birth, sex, level of education, myocardial infarction (I21–I23), hypertension (I10–I15), hyperlipidaemia (E78), diabetes (E10–E14), chronic obstructive pulmonary disease (J40–J44), cancer (C00–C97), obesity (E66), Raynaud’s phenomenon (I73.0), migraine (G43), cluster headache (G44.0), myocarditis (I40, I41), oesophageal disease (K20–K22), alcohol abuse (F10, Z71.4, Z72.1), cocaine abuse (F14) and tobacco use (F17, Z71.6, Z72.0). Familial HRs for VSA were calculated for relatives of individuals who had a diagnosis of VSA compared with relatives of individuals unaffected by VSA as the reference group. Subgroup analysis was performed by year of birth, recurrent hospitalisation for VSA (≥2 hospital admissions) and age difference between full-siblings. Two different sensitivity analyses were performed: (1) excluding all individuals who did not undergo coronary angiography (AF037) at the same hospital contact and (2) excluding those who had a coronary intervention (ie, angiography of coronary bypass grafts including internal mammary artery, coronary artery bypass grafting and percutaneous coronary intervention according to online supplemental table S1 during the index hospital contact for VSA. Spousal risk was also determined. Statistical significance was set at p < 0.05 and all tests were two-tailed. Data were analysed from December 2021 to February 2022 using SAS V.9.4 (SAS Institute).

10.1136/openhrt-2023-002504.supp1 Supplementary data

## Results

**Figure 1:**
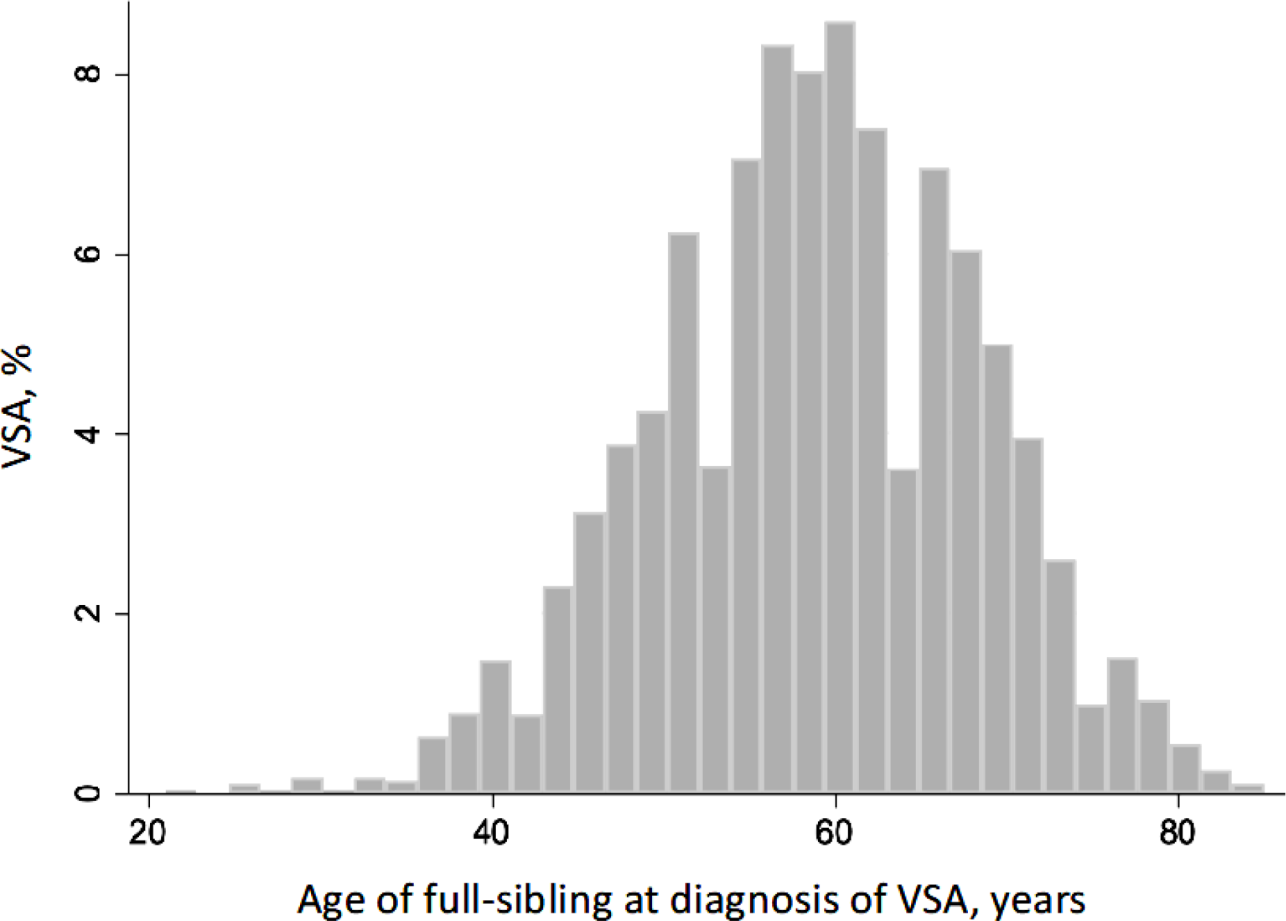
Age distribution at first-time diagnosis of vasospastic angina (VSA). Of 3461 individuals with VSA born between 1932 and 2018, the median age at onset was 59 (range, 21–85) years; the curve had a mean (SD) value of 59 (9.5) years.

The total study population included 5 764 770 full-siblings. Overall, 3461 (0.06%) unique individuals were diagnosed with VSA. Median age at first-time diagnosis was of disease 59 years (IQR 53–66) (figure 1). Individuals with a documented history of VSA were predominantly women and smokers, exhibiting a higher prevalence of comorbidities such as hypertension, dyslipidaemia, chronic obstructive pulmonary disease, obesity, diabetes, prior myocardial infarction, migraine, cancer and oesophageal disorders (table 1).

**Table 1:** Details of study population and stratified by documented history of vasospastic angina (VSA)

	Participants, no. (%)		
	All	Without VSA	With VSA
Overall	5 764 770	5 761 309 (99.94)	3461 (0.06)
Sex			
Male	2 954 982 (51.26)	2 953 757 (51.27)	1225 (35.39)
Female	2 809 788 (48.74)	2 807 552 (48.73)	2236 (64.61)
Year of birth, median (IQR) [range]	1974 (1955–1994) [1932–2018]	1974 (1955–1994) [1932–2018]	1946 (1941–1954) [1932–1990]
Age at end of follow-up, median (IQR) [range]	43 (24–62) [0–86]	43 (24–62) [0–86]	59 (53–66) [21–85]
Age at Prinzmetal onset, median (IQR) [range]	NA	NA	59 (53–66) [21–85]
Education			
Unknown or 1–9 years	692 193 (12.01)	691 197 (12.00)	996 (28.78)
10–11 years	2 422 567 (42.02)	2 420 999 (42.02)	1568 (45.30)
>11 years	2 650 010 (45.97)	2 649 113 (45.98)	879 (25.92)
Source			
Hospital discharge register	NA	NA	2218 (64.09)
Outpatient register	NA	NA	1243 (35.91)
Spouse			
Husband	NA	NA	1199 (39.38)
Wife	NA	NA	1846 (60.62)
Comorbidity			
Hypertension	640 698 (11.11)	638 405 (11.08)	2293 (66.25)
Hyperlipidaemia	222 912 (3.87)	221 306 (3.84)	1606 (46.40)
COPD	108 472 (1.88)	108 092 (1.88)	380 (10.98)
Obesity	163 979 (2.84)	163 688 (2.84)	291 (8.41)
Diabetes mellitus	254 726 (4.42)	253 996 (4.41)	730 (21.09)
Myocardial infarction	117 747 (2.04)	116 661 (2.02)	1086 (31.38)
Raynaud’s phenomenon	4216 (0.07)	4197 (0.07)	19 (0.55)
Migraine	111 908 (1.94)	111 731 (1.94)	177 (5.11)
Cluster headache (Horton syndrome)	4765 (0.08)	4759 (0.08)	6 (0.17)
Myocarditis	8420 (0.15)	8390 (0.15)	30 (0.87)
Malignancy	520 725 (9.03)	519 826 (9.02)	899 (25.98)
Oesophagal disease	185 985 (3.23)	185 427 (3.22)	558 (16.12)
Alcohol abuse	178 405 (3.09)	178 222 (3.09)	183 (5.29)
Cocaine abuse	2294 (0.04)	2292 (0.04)	2 (0.06)
Tobacco use	93 406 (1.62)	93 087 (1.62)	319 (9.22)

COPD, chronic obstructive pulmonary disease.

### Familial risk of VSA

Person-years, incidence rates and IRR according to documented history of VSA are presented in table 2. The incidence rate of VSA for those with an affected sibling was 0.31 (95% CI: 0.24 to 0.42) per 1000 person-years compared with 0.04 (95% CI: 0.04 to 0.04) per 1000 person-years for those without an affected sibling, yielding an IRR of 7.58 (95% CI: 5.71 to 10.07) (online supplemental file 2).

10.1136/openhrt-2023-002504.supp2 Supplementary data

**Table 2:** Risk of vasospastic angina (VSA) in siblings with and without an affected sibling

Variable	Person-year no.	Cases, no./ persons at risk, no.	Incidence rate, cases/1000 person-years	Incidence rate ratio (95% CI)	HR (95% CI)		
					Model 1	Model 2	Model 3
Sibling not affected	187 909 313	7801/10 136 643	0.04 (0.04–0.04)	1 (Reference)	1 (Reference)	1 (Reference)	1 (Reference)
Sibling affected	152 474	48/7849	0.31 (0.24–0.42)	7.58 (5.71 to 10.07)	7.64 (5.17 to 11.31)	3.39 (2.28 to 5.03)	2.56 (1.73 to 3.79)

Model 1 unadjusted. Model 2 adjusted for birth year, sex and educational attainment. Model 3 additionally adjusted for hypertension, hyperlipidaemia, COPD, obesity, diabetes mellitus, myocardial infarction, Raynaud’s phenomenon, migraine, cluster headache Horton syndrome, myocarditis, malignancy, oesophageal disease, alcohol abuse, cocaine abuse, tobacco use.

All calculations are based on double entry and 95% CI are calculated using robust SEs.

COPD, chronic obstructive pulmonary disease.

**Figure 2:**
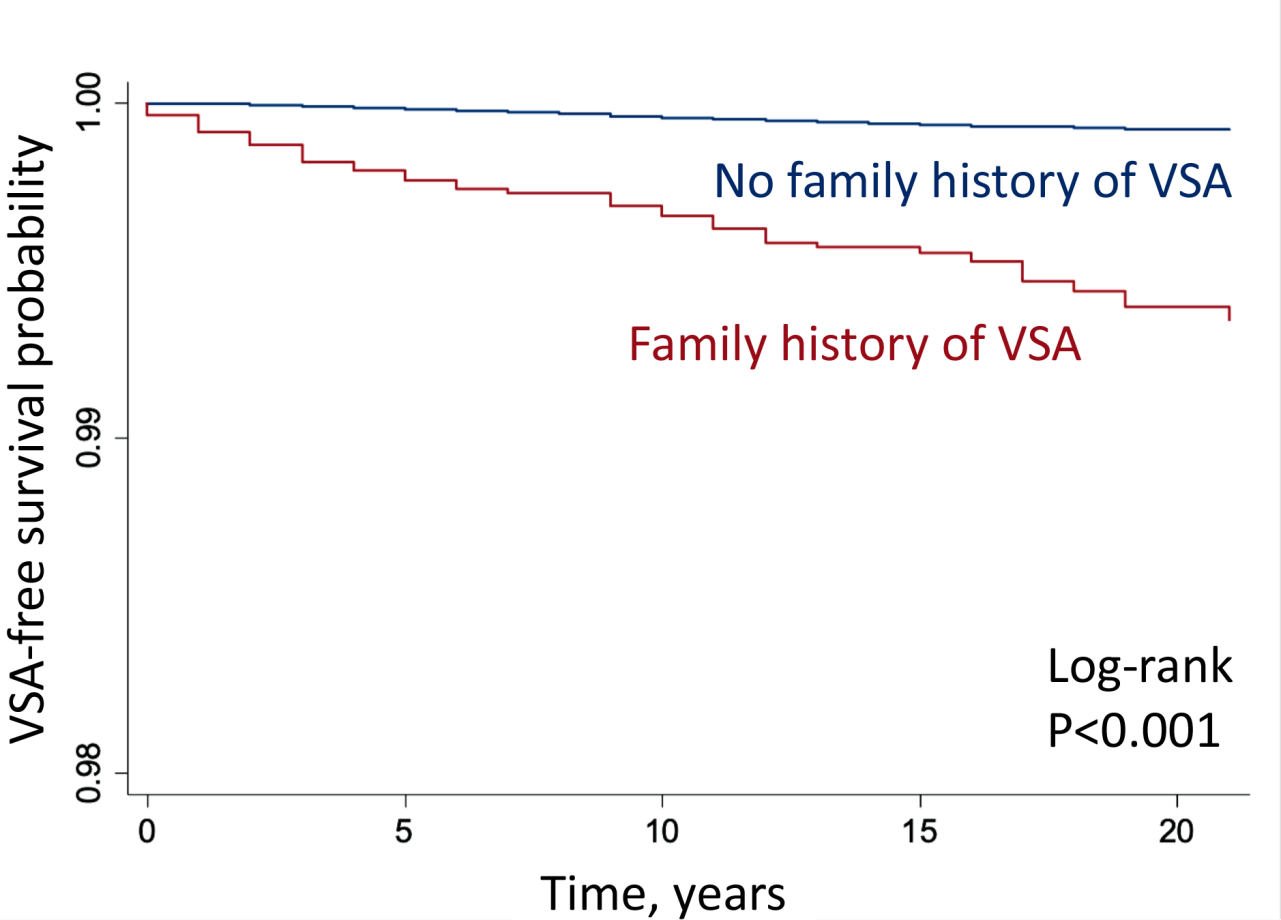
Vasospastic angina (VSA) survival curves with and without sibling history of VSA. Kaplan-Meier VSA-free survival estimates by family (ie, sibling) history.

Table 2 shows, in the crude Model 1, that the risk of VSA for siblings with an affected sibling was increased (HR: 7.64; 95% CI: 5.17 to 11.31). In the multivariable-adjusted Model 2, which also included birth year, sex and educational attainment, the familial HR for VSA was 3.39 (95% CI: 2.28 to 5.03). After additional adjustment for comorbidities in Model 3, the familial HR remained significantly increased (HR: 2.56; 95 % CI: 1.73 to 3.79) (online supplemental file 2). Kaplan-Meier curves according to the family history of VSA are presented in figure 2.

Family risk of VSA was consistent in subgroup analyses stratified by year of birth (online supplemental table S2) and recurrent VSA (online supplemental table S3). Individuals with recurrent VSA had higher familial risk (HR: 5.45; 95% CI: 2.80 to 10.62). Familial sibling risk was higher among younger (HR: 4.63; 95% CI: 2.12 to 10.14) than older (HR: 2.36; 95% CI: 1.54 to 3.63) individuals. In a sensitivity analysis limited to patients who underwent coronary angiography during their index hospital contact, the fully adjusted model produced a familial HR of 4.31 (95% CI: 0.62 to 30.15) (online supplemental table S4). When excluding patients undergoing myocardial revascularisation during the index hospital contact, the fully adjusted model yielded a familial HR of 2.52 (95% CI: 1.65 to 3.84) (online supplemental table S5).

We conducted two additional types of analyses to assess the extent of environmental sharing in the observed risks of VSA. First, we computed HRs for siblings based on age difference. The age difference had minimal impact on the results. Siblings with an age difference of less than 6 years exhibited an HR of 2.59 (95% CI: 1.59 to 4.21), while those with a difference of 6 years or more had an HR of 2.48 (95% CI: 1.26 to 4.90) (online supplemental table S6). Second, we estimated risks for spouses hospitalised for VSA. No increased risk of VSA was observed in spouses of affected individuals (HR: 0.63; 95% CI: 0.19 to 2.09) (online supplemental table S7).

In multivariate Cox regression analysis, family history of VSA, female sex, diabetes, obesity, hyperlipidaemia, history of myocardial infarction, tobacco use, oesophageal disease, history of myocarditis and alcohol abuse remained independent predictors of VSA in the fully adjusted model (online supplemental table S8).

## Discussion

In this nationwide family study, we identified signals suggesting a strong hereditary component in VSA with genetic resemblance, which provides valuable insights into the potential role of genetic factors in VSA and coronary vasomotor disorders development. The familial association was independent of traditional cardiovascular risk factors and clinically relevant comorbidities. The observed familial clustering of VSA highlights the importance of identifying factors that contribute to this aggregation, which include both genetic and non-genetic elements. Delving into these factors may provide valuable insights into VSA pathophysiology.

The higher familial risk among younger than older individuals argues for an important genetic contribution. Complex traits typically exhibit stronger inheritance at young ages.^31 32^ The observation of similar HRs among siblings with both small and large age differences further strengthens the argument for a genetic contribution. The lack of association among spouses also indicates that genetics might be more important than shared familial household environment. Numerous complex disorders typically exhibit ORs of approximately 2.^32^ Very strong environmental risk factors with complete correlation in exposure among siblings are necessary to explain the present findings.^21^

The increased prevalence of VSA among female patients observed in our study corroborates recent evidence from one of the largest and contemporary European cohort focusing on sex differences in coronary vasomotor dysfunction.^33^ This cohort, which examined patients with stable angina and unobstructed coronary arteries undergoing acetylcholine testing, revealed that women are more prone to conditions such as epicardial vasospasm and coronary microvascular dysfunction caused by microvascular spasms, and also exhibit greater sensitivity to acetylcholine compared with men. In addition, their finding that positive family history is more frequently identified in women than in men suggests potentially different genetic backgrounds.

The current understanding of VSA pathophysiology involves various factors, including endothelial dysfunction,^34^ smooth muscle hyper-reactivity,^22 35^ autonomic dysfunction^36 37^ and inflammation.^1 14 38^ Although the precise mechanisms leading to VSA are still not fully understood, our findings indicate that inheritable factors could play a significant role in its development. Additionally, our study highlights the importance of considering family history in the clinical evaluation of patients with suspected VSA. Clinicians should be aware of the increased risk of VSA in first-degree relatives of affected individuals, as this information may facilitate early identification and timely intervention in at-risk individuals. It is worth noting that, despite the strong heritable component observed in this study, non-genetic factors likely play a role in the development of VSA as well. Environmental factors, lifestyle and other modifiable risk factors should not be overlooked in the management of VSA.^39–41^ A comprehensive approach addressing both genetic and non-genetic factors is essential for the effective prevention and treatment of patients with coronary vasomotor disorders.

Family risk studies are instrumental in unveiling familial disease patterns and understanding genetic and environmental contributions to complex diseases like VSA. These studies help identify shared genetic factors among relatives, potentially serving as valuable biomarkers for early detection and disease prevention. Furthermore, family risk studies deliver a comprehensive view of the disease, considering the complex interplay between genetic, environmental and lifestyle factors. Genome-wide association studies (GWAS) offer a complementary approach to our family risk study on VSA. GWAS can help identify common genetic variants associated with VSA, providing further insights into the genetic architecture of the disease and potential therapeutic targets. Combining the results of family risk studies with GWAS findings may lead to a more complete understanding of genetic susceptibility and environmental factors that contribute to VSA development. In a small study recruiting 411 Japanese women with stable chest pain who underwent intracoronary acetylcholine provocation test, a novel genetic marker for coronary spasm, SNP rs10498345, was identified using a genome-wide single nucleotide polymorphism analysis. The SNP rs10498345 may hold clinical significance as it was found more strongly associated with VSA than cigarette smoking.^42^

Further research is needed to validate and expand on our findings and identify specific genetic factors and pathways involved in the pathophysiology of VSA that may help reveal novel therapeutic targets, guide the development of personalised treatment approaches and targeted screening programmes for high-risk populations. This research could include larger, multicentre and multiethnic studies to identify common and population-specific genetic factors, as well as investigations into gene–environment interactions and the potential influence of epigenetic factors on VSA risk.

### Strengths and limitations

The large size and nationwide coverage of the study sample is a key strength. Other important strengths include the use of validated national hospital discharge data,^23–27^ which allows for the elimination of recall bias, and the capability of controlling for major confounders, such as age, sex, cardiovascular risk factors, cancer, smoking, myocarditis, oesophageal disorders,^43^ Raynaud’s phenomenon and migraine headaches.^44^ Further, Swedish registers such as the Multigeneration Register and the Swedish Hospital Discharge Register have high coverage and high data validity.^24 45 46^

A limitation is the lack of molecular biomarkers, ethnicity, genetic and genomic information. For instance, in familial studies, there is always the issue of assurance of paternity.^47^ However, a recent Swedish study shows that among offspring born 1950 and later the frequency of misattributed paternity in Sweden is low (1.7%) and has decreased to 1%. Another limitation is that the study was limited to Sweden, which may limit the generalisability of the findings to other populations and ethnicities. In addition, the observational nature of the study does not allow for establishing causality between familial risk and VSA development. Other limitations include the evolving diagnostic criteria for VSA over time, the inconsistent application of diagnostic techniques to demonstrate coronary vasospasm and the lack of direct access to original diagnostic investigations. COVADIS criteria were proposed first in 2013 and most of the database-derived diagnoses were established before this date. However, after the exclusion of cases where coronary angiography was not performed during the index hospital admission, the association between familial relationship and VSA diagnosis was even stronger.

## Conclusions

In this nationwide family study, we identified strong hereditary components in VSA with genetic resemblance. The familial association was independent of cardiovascular risk factors and clinically relevant comorbidities. Our results indicate that VSA tends to cluster in families, implying that uncovering the sources of this familial aggregation, including both genetic and non-genetic factors, could be valuable. This knowledge has relevant implications for the clinical evaluation of patients with suspected VSA, risk assessment of family members and the development of personalised prevention and treatment strategies. Additionally, the findings highlight the need for continued research to elucidate the genetic and non-genetic factors contributing to VSA.

## Data Availability

Data are available upon reasonable request.
