# Supplementary material for: Familial risk of vasospastic angina: a nationwide family study in Sweden
Source: Open Heart. 2023 Dec 6;10(2):e002504. doi: 10.1136/openhrt-2023-002504 (PMC10711886; doi:10.1136/openhrt-2023-002504)
Supplement: Supplementary data [file openhrt-2023-002504supp001.pdf]

Supplementary Tables S1-S8

Table S1. Exclusion codes used in Table S4 for myocardial revascularization

	Surgery and medical codes
CABG	FNA00, FNA10, FNA20, FNA96, FNB00, FNB20, FNB96, FNC10, FNC20, FNC30, FNC40, FNC50, FNC60, FNC96, FND10, FND20, FND96, FNE00, FNE10, FNE20, FNE96
Other coronary interventions, including PCI	FNF00, FNF10, FNF20, FNF30, FNF96, FNG00, FNG02, FNG05, FNG10, FNG20, FNG22, FNG30, FNG96, FNH00, FNH10, FNH20, FNH96, FNJ00, FNJ02, FNJ10, FNJ12, FNJ96, FNK00, FNK10, FNK20, FNK96, FNW96, FNW98

Table S2. Familial risk among of vasospastic angina (VSA) stratified by year of birth.

Year of birth	Variable	Person-years, No.	Cases, No./Persons at risk, No.	Incidence rate, cases/1000 person-years	Incidence rate ratio (95%CI)	HR(95% CI) **Model 1**	**Model 2**	**Model 3**
< 1955	Sibling not affected	58166669	6165/2988167	0.11 (0.10-0.11)	1 [Reference]	1 [Reference]	1 [Reference]	1 [Reference]
	Sibling affected	111761	38/5864	0.34 (0.25-0.47)	3.21 (2.33-4.41)	3.20 (2.08-4.92)	3.05 (1.98-4.70)	2.36 (1.54-3.63)
≥ 1955	Sibling not affected	129742644	1636/7148476	0.01 (0.01-0.01)	1 [Reference]	1 [Reference]	1 [Reference]	1 [Reference]
	Sibling affected	40713	10/1985	0.24 (0.13-0.46)	19.48 (10.46-36.27)	18.99 (8.77-41.16)	6.31 (2.92-13.64)	4.63 (2.12-10.14)

Abbreviations: HR=hazard ratio, CI=confidence interval.
Model 1 unadjusted. Model 2 adjusted for birth year, sex and educational attainment. Model 3 additionally adjusted for Hypertension, Hyperlipidemia, COPD, Obesity, Diabetes mellitus, Myocardial infarction, Raynaud´s phenomenon, Migraine, Cluster headache Horton syndrome, Myocarditis, Malignancy, Oesophageal disease, Alcohol abuse, Cocaine abuse, Tobacco use.
All calculations are based on double entry and 95% CI are calculated using robust standard errors.

Table S3. Familial risk of recurrent episodes (≥2) of vasospastic angina (VSA) in the national patient register (NPR).

Variable	Person-years, No.	Cases, No./Persons at risk, No.	Incidence rate, cases/1000 person-years	Incidence rate ratio (95%CI)	HR(95% CI) **Model 1**	**Model 2**	**Model 3**
Sibling not affected	188000518	3119/10141357	0.02 (0.02-0.02)	1 [Reference]	1 [Reference]	1 [Reference]	1 [Reference]
Sibling affected	61269	16/3135	0.26 (0.16-0.43)	15.74 (9.63-25.73)	15.86 (8.01-31.40)	7.54 (3.79-14.99)	5.45 (2.80-10.62)

Abbreviations: HR=hazard ratio, CI=confidence interval.
Model 1 unadjusted. Model 2 adjusted for birth year, sex and educational attainment. Model 3 additionally adjusted for Hypertension, Hyperlipidemia, COPD, Obesity, Diabetes mellitus, Myocardial infarction, Raynaud´s phenomenon, Migraine, Cluster headache Horton syndrome, Myocarditis, Malignancy, Esophageal disease, Alcohol abuse, Cocaine abuse, Tobacco use.
All calculations are based on double entry and 95% CI are calculated using robust standard errors.

Table S4. Familial risk of vasospastic angina (VSA) defined by diagnosis of VSA and the occurrence of coronary angiography (AF037) during the same date in the national patient register (NPR).

Variable	Person-years, No.	Cases, No./Persons at risk, No.	Incidence rate, cases/1000 person-years	Incidence rate ratio (95%CI)	HR(95% CI) **Model 1**	**Model 2**	**Model 3**
Sibling not affected	188036507	1286/10143204	0.01 (0.01-0.01)	1 [Reference]	1 [Reference]	1 [Reference]	1 [Reference]
Sibling affected	25280	2/1288	0.08 (0.02-0.32)	11.57 (2.89-46.30)	11.38 (1.64-78.89)	6.06 (0.86-42.90)	4.31 (0.62-30.15)

Abbreviations: HR=hazard ratio, CI=confidence interval.
Model 1 unadjusted. Model 2 adjusted for birth year, sex and educational attainment. Model 3 additionally adjusted for Hypertension, Hyperlipidemia, COPD, Obesity, Diabetes mellitus, Myocardial infarction, Raynaud´s phenomenon, Migraine, Cluster headache Horton syndrome, Myocarditis, Malignancy, Esophageal disease, Alcohol abuse, Cocaine abuse, Tobacco use.
All calculations are based on double entry and 95% CI are calculated using robust standard errors.

Table S5. Sibling risk of vasospastic angina (VSA) after exclusion of VSA cases undergoing myocardial revascularization according to Table S1 at the same date of diagnosis

Variable	Person-years, No.	Cases, No./Persons at risk, No.	Incidence rate, cases/1000 person-years	Incidence rate ratio (95%CI)	HR(95% CI) **Model 1**	**Model 2**	**Model 3**
Sibling not affected	187905469	7424/10136266	0.04 (0.04-0.04)	1 [Reference]	1 [Reference]	1 [Reference]	1 [Reference]
Sibling affected	145115	42/7466	0.29 (0.21-0.39)	7.33 (5.41-9.92)	7.37 (4.84-11.23)	3.28 (2.15-5.01)	2.52 (1.65-3.84)

Abbreviations: HR=hazard ratio, CI=confidence interval.

Model 1 unadjusted. Model 2 adjusted for birth year, sex and educational attainment. Model 3 additionally adjusted for Hypertension, Hyperlipidemia, COPD, Obesity, Diabetes mellitus, Myocardial infarction, Raynaud´s phenomenon, Migraine, Cluster headache Horton syndrome, Myocarditis, Malignancy, Esophageal disease, Alcohol abuse, Cocaine abuse, Tobacco use. All calculations are based on double entry and 95% CI are calculated using robust standard errors.

Table S6. **Sibling risk of vasospastic angina (VSA) stratified by age difference between siblings.**

Age difference	Variable	Person-years, No.	Cases, No./Persons at risk, No.	Incidence rate, cases/1000 person-years	Incidence rate ratio (95%CI)	HR(95% CI) **Model 1**	**Model 2**	**Model 3**
< 6 years	Sibling not affected	129571172	4933/ 7111605	0.04 (0.04-0.04)	1 [Reference]	1 [Reference]	1 [Reference]	1 [Reference]
	Sibling affected	96110	32/4965	0.33 (0.24-0.47)	8.75 (6.18-12.38)	8.79 (5.40-14.29)	3.36 (2.06-5.47)	2.59 (1.59-4.21)
≥ 6 years	Sibling not affected	58338141	2868/ 3025038	0.05 (0.05-0.05)	1 [Reference]	1 [Reference]	1 [Reference]	1 [Reference]
	Sibling affected	56364	16/2884	0.28 (0.17-0.46)	5.77 (3.53-9.44)	5.79 (2.92-11.50)	3.42 (1.72-6.78)	2.48 (1.26-4.90)

Abbreviations: HR=hazard ratio, CI=confidence interval.

Model 1 unadjusted. Model 2 adjusted for birth year, sex and educational attainment. Model 3 additionally adjusted for Hypertension, Hyperlipidemia, COPD, Obesity, Diabetes mellitus, Myocardial infarction, Raynaud´s phenomenon, Migraine, Cluster headache Horton syndrome, Myocarditis, Malignancy, Oesophageal disease, Alcohol abuse, Cocaine abuse, Tobacco use. All calculations are based on double entry and 95% CI are calculated using robust standard errors.

Table S7. **Familial risk of vasospastic angina (VSA) in spouses of affected individuals.**

Variable	Person-years, No.	Cases, No./Persons at risk, No.	Incidence rate, cases/1000 person-years	Incidence rate ratio (95%CI)	HR(95% CI) **Model 1**	**Model 2**	**Model 3**
Spouse not affected	187933547	7841/ 10136643	0.04 (0.04-0.04)	1 [Reference]	1 [Reference]	1 [Reference]	1 [Reference]
Spouse affected	128240	8/ 6529	0.06 (0.03-0.12)	1.50 (0.75-2.99)	1.51 (0.46-4.99)	0.69 (0.21-2.30)	0.63 (0.19-2.09)

Abbreviations: HR=hazard ratio, CI=confidence interval.
Model 1 unadjusted. Model 2 adjusted for birth year, sex and educational attainment. Model 3 additionally adjusted for Hypertension, Hyperlipidemia, COPD, Obesity, Diabetes mellitus, Myocardial infarction, Raynaud´s phenomenon, Migraine, Cluster headache Horton syndrome, Myocarditis, Malignancy, Oesophageal disease, Alcohol abuse, Cocaine abuse, Tobacco use.
All calculations are based on double entry and 95% CI are calculated using robust standard errors.

Table S8. Crude and multivariate Cox proportional hazard models.

		HR (95% CI)		
	**Reference**	**Model 1**	**Model 2**	**Model 3**
**Sibling affected**	No	7.64 (5.17-11.31)	3.39 (2.28-5.03)	2.56 (1.73-3.79)
**Female Sex**	Male	1.87 (1.72-2.03)	1.80 (1.66-1.96)	2.41 (2.13-2.63)
**Year of born**		0.93 (0.92-0.93)	0.93 (0.93-0.93)	0.96 (0.95-0.96)
**Education**				
10-11 years	Unknown or 1-9 years	0.51 (0.46-0.56)	1.04 (0.94-1.15)	1.11 (1.01-1.23)
>11 years		0.29 (0.26-0.32)	0.85 (0.76-0.96)	1.08 (0.96-1.21)
**Comorbidity**				
Hypertension	No	12.10 (11.11-13.17)		2.38 (2.09-2.71)
Hyperlipidemia	No	16.39 (15.12-17.77)		3.52 (3.13-3.95)
COPD	No	5.20 (4.57-5.91)		1.09 (0.95-1.25)
Obesity	No	2.89 (2.49-3.34)		1.25 (1.08-1.46)
Diabetes mellitus	No	4.92 (4.47-5.43)		0.93 (0.83-1.03)
Myocardial infarction	No	17.00 (15.59-18.55)		3.59 (3.20-4.02)
Raynaud´s phenomenon	No	5.94 (3.44-10.24)		3.10 (1.78-5.40)
Migraine	No	2.41 (2.02-2.87)		2.29 (1.91-2.73)
Cluster headache Horton syndrome	No	1.69 (0.64-4.44)		1.08 (0.41-2.87)
Myocarditis	No	5.38 (3.46-8.39)		3.16 (2.05-4.87)
Malignancy	No	2.98 (2.72-3.26)		1.04 (0.94-1.14)
Esophageal disease	No	4.98 (4.47-5.56)		2.02 (1.81-2.27)
Alcohol abuse	No	1.50 (1.26-1.80)		1.27 (1.06-1.53)
Cocaine abuse	No	1.60 (0.32-7.97)		2.09 (0.42-10.42)
Tobacco use	No	4.74 (4.13-5.45)		1.32 (1.14-1.54)

Abbreviations: HR=hazard ratio, CI=confidence interval. Model 1 crude. Model 2 Prinzmetal adjusted for birth year, sex and educational attainment. Model 3 additionally adjusted for Hypertension, Hyperlipidemia, COPD, Obesity, Diabetes mellitus, Myocardial infarction, Raynaud´s phenomenon, Migraine, Cluster headache Horton syndrome, Myocarditis, Malignancy, Esophageal disease, Alcohol abuse, Cocaine abuse, Tobacco use. All calculations are based on double entry and 95% CI are calculated using robust standard errors.
