# Supplementary material for: Familial risk of vasospastic angina: a nationwide family study in Sweden
Source: Open Heart. 2023 Dec 6;10(2):e002504. doi: 10.1136/openhrt-2023-002504 (PMC10711886; doi:10.1136/openhrt-2023-002504)

Familial Risk of Vasospastic Angina: a Nationwide Family Study in Sweden

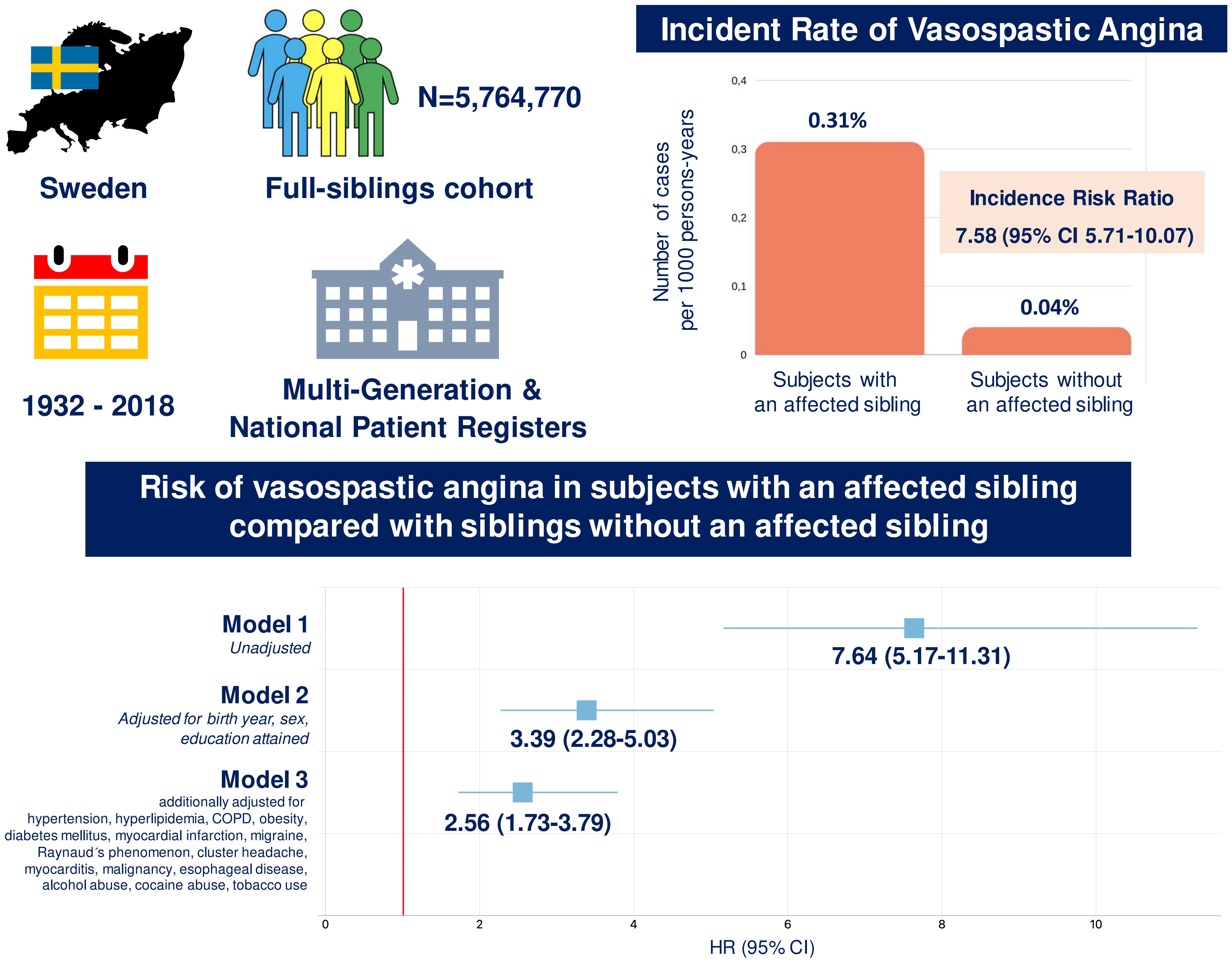

Supplement: Supplementary data [file openhrt-2023-002504supp002.pdf]
